# Impact of hearing impairment on cognitive performance

**DOI:** 10.1016/j.bjorl.2024.101521

**Published:** 2024-11-05

**Authors:** Pedro Ivo Machado Pires de Araújo, Pauliana Lamounier e Silva Duarte, Hugo Valter Lisboa Ramos, Claudiney Cândido Costa, Isabela Gomes Maldi, Lucas da Silva Braz, Norma de Oliveira Penido

**Affiliations:** a Programa de Cooperação Institucional CRER - Escola Paulista de Medicina (EPM), Universidade Federal de São Paulo (UNIFESP), São Paulo, SP, Brazil; b CRER Hospital, São Paulo, SP, Brazil; c Escola Paulista de Medicina (EPM), Universidade Federal de São Paulo (UNIFESP), São Paulo, SP, Brazil

**Keywords:** Hearing impairment, Cognition, Cognitive impairment, Dementia, Mini-mental state examination

## Abstract

**Objective:**

This study aims to evaluate the cognitive performance in adults with hearing loss and to identify associations between clinical characteristics of hearing loss and cognitive outcomes.

**Methods:**

In this cross-sectional analytical observational study, adults with hearing loss underwent the Mini-Mental State Examination (MMSE), following the collection of their clinical and audiometric data.

**Results:**

Among 134 evaluated individuals, a majority reported a progressive onset (91.04%) and bilateral nature (87.31%) of hearing loss, with moderate hearing loss being the most common (41.04%). Sensorineural hearing loss was prevalent in 76.12% of cases, with presbycusis identified as a primary etiology in 37.31%. Comorbidities were reported in 61.19% of participants, with 16.42% using benzodiazepines or antidepressants regularly. Symptoms included imbalance (33.58%), vertigo (42.54%), and tinnitus (73.88%). Notably, a sudden onset of hearing loss and imbalance complaints were linked to a higher likelihood of subnormal MMSE performance. Analysis revealed varied cognitive domain performances associated with different clinical characteristics of hearing loss.

**Conclusion:**

Various aspects of hearing loss, such as bilateral and sensorineural types, and the presence of symptoms like tinnitus and vertigo, significantly influence cognitive performance. Specifically, sudden onset hearing loss and imbalance complaints are associated with poorer overall cognitive outcomes in the MMSE. These findings underscore the importance of considering the diverse impacts of hearing loss characteristics on cognitive functions.

**Level of evidence:**

Level 4.

## Highlights

• Characteristics inherent to hearing loss associated with performance on the MMSE.
• Sudden onset hearing loss tied to lower MMSE scores.
• Link found between hearing loss type and cognitive impact in adults.

## Introduction

Hearing loss, as defined by the World Health Organization (WHO), is a growing public health concern, affecting over 5% of the global population, including a significant proportion of those over 65 years old [1]. The increase in hearing loss cases is paralleled by a rise in cognitive issues and dementia, highlighting a need for in-depth research into their interrelation.

Cognitive impairment varies from normal age-related changes to severe conditions like dementia. With the global prevalence of dementia expected to rise significantly by 2050 [2], [3], [4], understanding the link between hearing loss and cognitive decline is increasingly important.

Studies show that hearing loss could be a modifiable risk factor for cognitive decline [2], [5], [6], [7], [8], [9], [10]. Potential connections between hearing loss and cognitive impairment include sensory deprivation, cognitive overload, and social isolation. Moreover, global neural degradation due to aging could be a common cause for both phenomena [11], [12]. Despite this, the exact mechanisms underlying the hearing loss-cognitive impairment relationship remain unclear.

The economic impact of hearing loss and cognitive impairment is substantial, with the WHO estimating billions of US dollars in annual costs [3], [13]. This underlines the importance of understanding the association between these two conditions for public health policies and interventions.

Several studies have explored the association between hearing loss and cognitive decline, revealing a complex and multifaceted relationship.[14] For instance, a prospective cohort of individuals who were followed-up at the iconic Framingham Heart Study found association between hearing loss and worsened Mini Mental State Examination (MMSE) performance [15]. Other studies have identified factors like social isolation and sedentary lifestyle as mediating the relationship between hearing loss and cognitive decline [16], [17], [18].

However, challenges in understanding this relationship persist. For example, sensory impairment might influence performance in cognitive tests, potentially skewing results [16], [19], [20]. Additionally, various pathophysiological theories propose different mechanisms for the hearing loss-cognition connection, such as sensory deprivation altering brain structure and function, or cognitive demands compensating for poor sensory input [11], [21], [22].

In summary, the increasing prevalence of hearing loss and its potential impact on cognitive performance, especially among older adults, highlights a pressing need for further research. This study aims to contribute to this field by assessing the cognitive performance of adults with hearing loss and exploring associations between clinical features of hearing loss and cognitive outcomes.

## Methods

The study was a cross-sectional analytical observational clinical survey using a questionnaire. It took place at the otolaryngology outpatient center of CRER (Centro Estadual de Reabilitação e Readaptação Dr. Henrique Santillo) Hospital in Goiânia City, Goiás State, Brazil, from January 2022 to February 2023.

We used a convenience sample of 134 adult volunteers from the hearing aids outpatient center of CRER Hospital, who had not yet begun using hearing aids. The sample size was determined based on the global prevalence of clinically relevant deafness [13], with a confidence interval of 95% and a margin of error of 4% (half-width of the 95% Confidence Interval).

Participants were included if they were over 18 years old, agreed voluntarily to the study, and had a medical diagnosis of unilateral or bilateral hearing loss with a recommendation for hearing aids. Those already using hearing aids or other hearing rehabilitation devices, and those with a prior neurocognitive disorder diagnosis, were excluded.

During outpatient visits, participants provided sociodemographic data (age, gender, ethnicity, and schooling) and clinical data (comorbidities like hypertension, diabetes, or dyslipidemia; smoking history; use of benzodiazepines or antidepressants; symptoms of tinnitus; complaints such of vertigo or imbalance; noise exposure history; and otologic surgery history). Data regarding the characteristics of hearing loss, including laterality, duration (hypoacusis time, in years, reported by the individual, according to their own perception), onset type, and etiology, were also collected from medical reports.

Participants' most recent audiometric exams provided data on bone conduction and airway pure tone thresholds across various frequencies. All participants had undergone a pure tone audiometry in a cabin of dimensions 2 × 2 m, using the same Interacoustics AC 33 audiometer. Hearing loss was classified according to WHO standards: normal (< 20 dB), mild (20 < 35 dB), moderate (35 < 50 dB), moderately severe (50 < 65 dB), severe (65 < 80 dB), profound (80 < 95 dB), and complete (95 dB or greater) [23].

The MMSE, validated in Brazilian Portuguese [24], was used to assess cognitive function immediately after clinical and audiometric data collection. The MMSE evaluates six cognitive domains with a total score range from 0 to 30. Its application was carried out by trained doctors. Participants with severe, profound, or complete hearing loss were encouraged to read the instructions to respond to each step of the test. The scores were classified as “normal” or “below normal” based on schooling level [24].

Poisson regression with robust variance was used for the statistical analysis. This method helped to identify factors associated with the occurrence below-normal general MMSE scores based on education level (dichotomous outcome), using Prevalence Ratio (PR) as the measure of effect. It was also used to identify factors associated with the number of questions answered correctly in each cognitive domain, using the average rate of correct responses as the measure of effect.

The analysis included both bivariate and multivariate stages, considering various independent variables (hearing loss laterality, time onset, degree and type) and potential confounders (age, comorbidities, smoking history, medication use, and these symptoms: imbalance, vertigo and tinnitus). The final model included variables with a significance level of *p* < 0.05. Multi-collinearity between variables was assessed, and the SAS software v. 9.4 was used for all analyses.

The study was approved by the Research Ethics Committee of Federal University of São Paulo (UNIFESP) (approval opinion n. 5.555.585). All participants signed informed consent forms before inclusion in the study.

## Results

### Sample descriptive results

The sample comprised 134 individuals who carried hearing loss and who were subjected to MMSE. In total, 53% of this number belonged to the female sex. Most participants declared themselves white (52%) and 13% of them declared themselves illiterate.

**Fig. 1:**
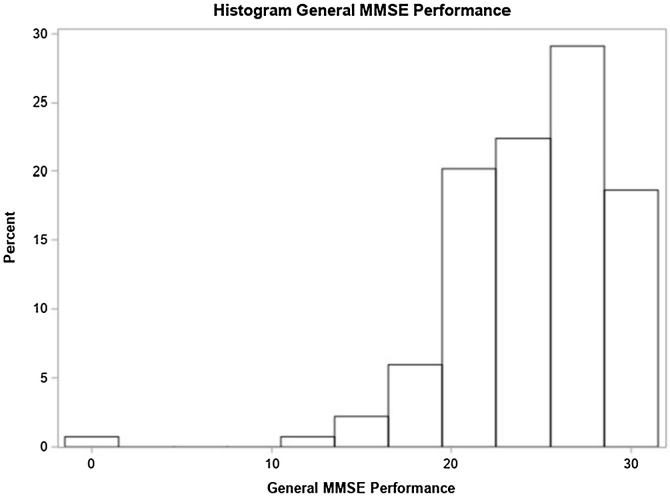
General MMSE performance.

Most participants (87%) carried bilateral hearing loss and most cases (48%) had unknown hearing loss etiology. Most participants (91%) presented progressive hearing loss and (61%) had, at least, one comorbidity (Systemic Arterial Hypertension ‒ SAH), diabetes mellitus or dyslipidemia). In total, 80% of participants were not smokers and only 16% of participants used benzodiazepines or antidepressants. Complaints of imbalance were present in 34% of individuals; vertigo in 43%; and tinnitus in 74% of the sample. Moderate hearing loss was the most frequent degree of it (41%). There was no record of conductive hearing loss in the sample, 24% of participants presented the mixed type of it and 76% of the sample were in the sensorineural-type group. Only 14% of participants stated that their hearing condition had affected their test understanding (Table 1). The distribution of overall performance scores on the MMSE is shown in Fig. 1.

**Table 1:** Demographic and clinical features.

Variable	Frequency (n = 134)	Percentage
Gender		
Female	71	52.99
Male	63	47.01
Age		
18‒39	15	11.19
40‒59	38	28.36
60‒79	64	47.76
≥ 80	17	12.69
Ethnicity		
Asian	2	1.49
White	70	52.24
Black	8	5.97
Brown	54	40.30
Schooling level		
Illiterate	18	13.43
1 to 4 schooling years	35	26.12
5 to 8 schooling years	35	26.12
9 to 11 schooling years	18	13.43
≥ 12 schooling years	28	20.90
Hearing loss laterality		
Bilateral	117	87.31
Unilateral	17	12.69
Etiology		
Unknown	64	47.76
Presbycusis	50	37.31
Chronic otitis media	9	6.72
Others	11	8.21
Hearing loss onset		
Progressive	122	91.04
Sudden	12	8.96
Comorbidities (SAH, Diabetes Mellitus or Dyslipidemia)		
No	52	38.81
Yes	82	61.19
Smoking		
Smoker or former smoker	27	20.15
Non-smoker	107	79.85
Use of medication (benzodiazepines or antidepressants)		
No	112	83.58
Yes	22	16.42

### Multivariate analysis – general MMSE performance

**Fig. 2:**
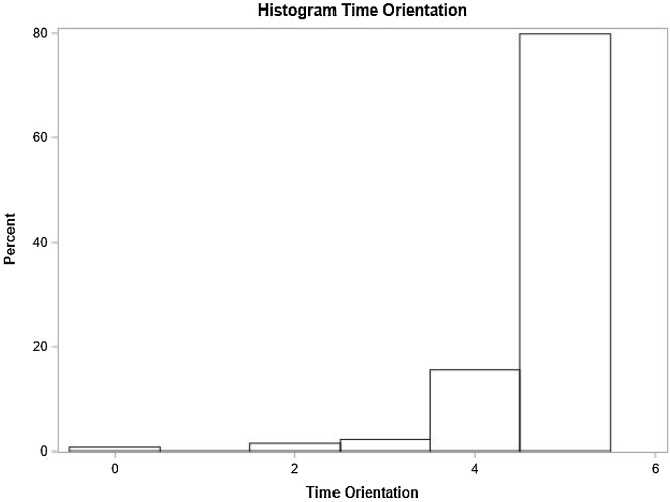
Time orientation domain performance.

**Fig. 3:**
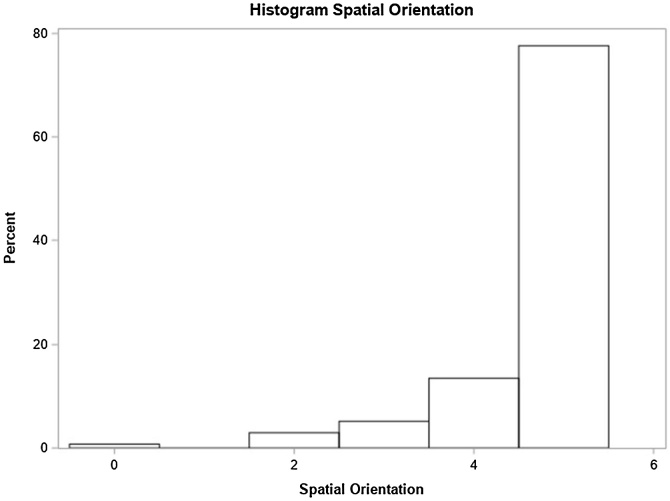
Spatial orientation domain performance.

**Fig. 4:**
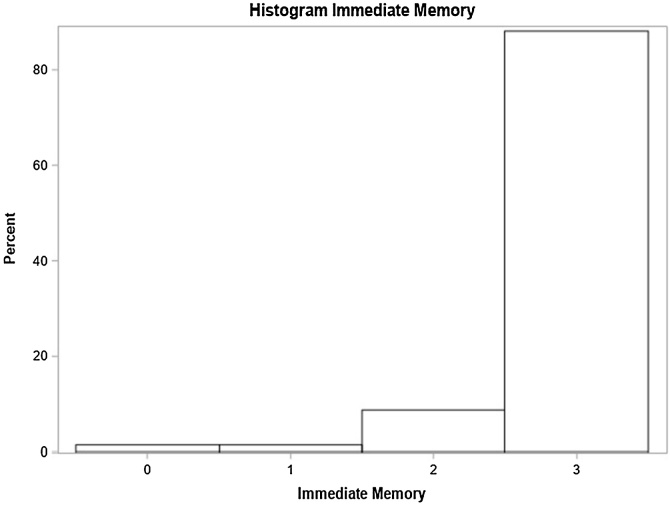
Immediate memory domain performance.

**Fig. 5:**
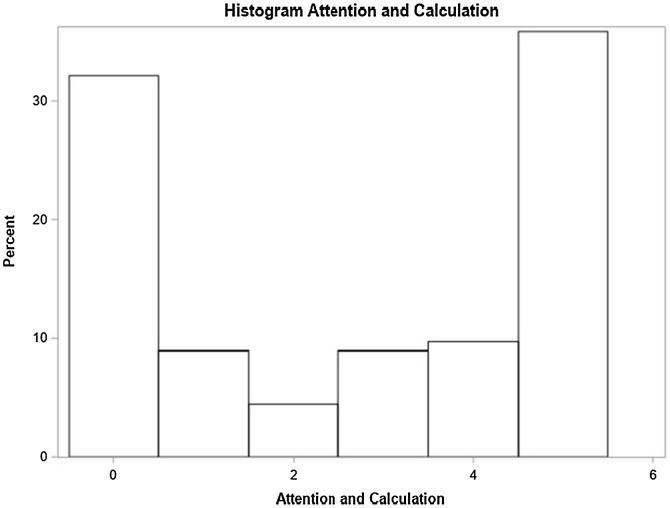
Attention and calculation domain performance.

**Fig. 6:**
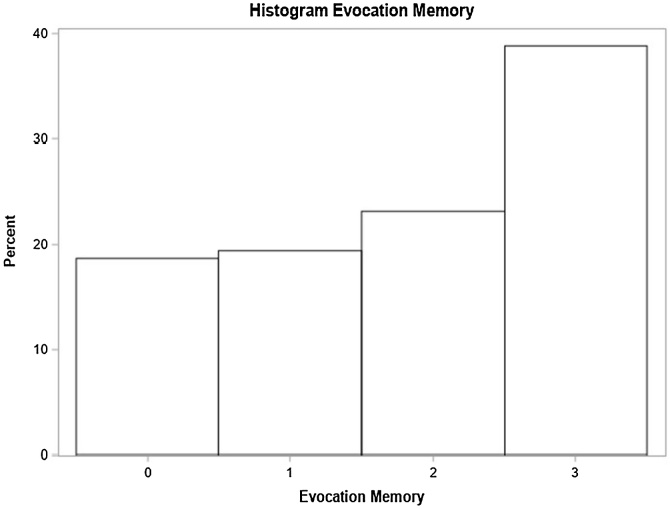
Evocation memory domain performance.

**Fig. 7:**
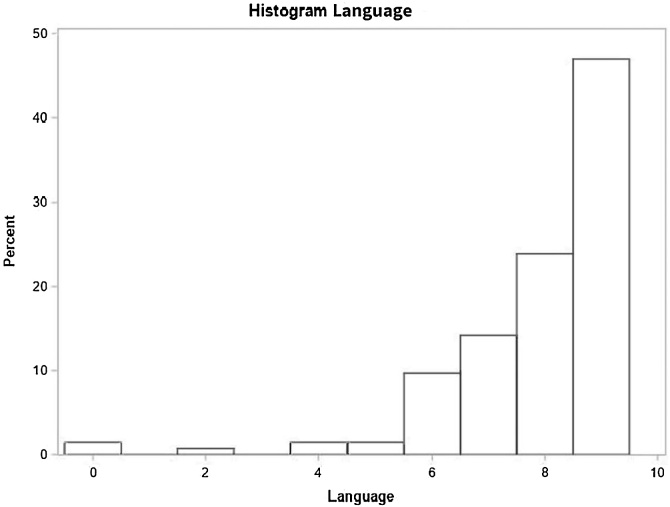
Language domain performance.

In the multivariate model, sudden hearing loss onset and balance issues were significant factors. Individuals with sudden hearing loss had a 53% higher chance of below-normal general MMSE scores based on education level compared to those with progressive loss. Those with balance issues showed a 43% higher prevalence of below-normal scores (Table 2). The distribution of scores in each of these specific domains is shown in Fig. 2, Fig. 3, Fig. 4, Fig. 5, Fig. 6, Fig. 7.

**Table 2:** Distribution of study variables based on gross and adjusted prevalence ratio, according to Poisson regression model with robust and adjusted variance and their respective 95% Confidence Intervals recorded for general MMSE score below normal, based on schooling (n = 134).

	Non-adjusted prevalence ratio (PR)		Adjusted Prevalence Ratio (PR)	
Variable	PR (95% CI)	*p*‒value	PR (95% CI)	*p*-value
Age	1.00 (0.99, 1.01)	0.7500	‒	‒
At least one comorbidity (SAH, DM or Dyslipidemia)		0.5982	‒	‒
No	1	‒	‒	‒
Yes	1.09 (0.80, 1.48)	0.5982	‒	‒
Smoker		0.7603	‒	‒
No	1	‒	‒	‒
Yes	1.06 (0.74, 1.51)	0.7603	‒	‒
Use of at least one medication (Benzodiazepine, Antidepressants)		0.1847	‒	‒
No	1	‒	‒	‒
Yes	1.25 (0.90, 1.74)	0.1847	‒	‒
Lack of balance		0.0112		0.0167^a^
No	1	‒	1	‒
Yes	1.44 (1.09, 1.90)	0.0112	1.43 (1.07, 1.92)	0.0167^a^
Vertigo		0.0431	‒	‒
No	1	‒	‒	‒
Yes	1.35 (1.01, 1.81)	0.0431	‒	‒
Tinnitus		0.1611	‒	‒
No	1	‒	‒	‒
Yes	1.33 (0.89, 1.97)	0.1611	‒	‒
Hearing loss degree		0.4788		0.7097
Normal or light	0.77 (0.49; 1.20)	0.2434	0.83 (0.54; 1.28)	0.4090
Moderate or moderately severe	1	‒	1	‒
Severe or complete	0.89 (0.54; 1.46)	0.6352	1.00 (0.48; 2.08)	0.9922
Laterality		0.4325		0.2385
Unilateral	0.81 (0.48; 1.37)	0.4325	0.66 (0.33; 1.32)	0.2385
Bilateral	1	‒	1	‒
Hypoacusis time (years)	1.00 (0.98, 1.01)	0.6298	0.99 (0.98, 1.01)	0.5028
Hearing loss onset		0.4146		0.0468^a^
Progressive	1	‒	1	‒
Sudden	1.20 (0.78, 1.84)	0.4146	1.53 (1.00, 2.36)	0.0468^a^
Hearing loss type		0.8613		0.2837
Mixed	1.03 (0.73, 1.45)	0.8613	1.23 (0.84, 1.79)	0.2837
Sensorineural	1	‒	1	‒

a *p* < 0.05.

### Multivariate analysis – MMSE cognitive domains

Individuals’ scores recorded for each cognitive domain forming MMSE were analyzed, namely: time orientation, spatial orientation, immediate memory, attention and calculation, evocation memory and language.

### Time orientation

Unilateral hearing loss, absence of comorbidities, and smoking status significantly influenced time orientation scores. Individuals with unilateral hearing loss performed 7% better than those with bilateral loss. Those without comorbidities and smokers also showed better performance (Table 3).

**Table 3:** Distribution of study variables according to the crude and adjusted average rate as per the Poisson regression model with robust variance and their respective 95% confidence intervals, for the number of correct responses in temporal orientation (n = 134).

Average rate ‒ time orientation				
Variable	Non-adjusted (95% CI)	*p-*value	Adjusted (95% CI)	*p*-value
Age	0.99 (0.98, 1.00)	0.0260	‒	‒
At least one comorbidity (SAH, DM or Dyslipidemia)		0.0496		0.0410^a^
No	1	‒	1	‒
Yes	0.96 (0.91;1.00)	0.0496	0.95 (0.90; 1.00)	0.0410^a^
Smoker		0.0907	‒	0.0482
No	1	‒	1	‒
Yes	1.04 (0.99, 1,08)	0.0907	1.05 (1.00, 1.10)	0.0482^a^
Use of at least one medication (Benzodiazepine, Antidepressants)		0.4926	‒	‒
No	1	‒	‒	‒
Yes	0.98 (0.92; 1.04)	0.4926	‒	‒
Lack of balance		0.1755	‒	‒
No	1	0.1755	‒	‒
Yes	0.96 (0.91, 1.02)	‒	‒	‒
Vertigo		0.9662	‒	‒
No	1	‒	‒	‒
Yes	1.00 (0.95, 1.05)	0.9662	‒	‒
Tinnitus		0.7750	‒	‒
No	1	0.7750	‒	‒
Yes	0.99 (0.94, 1.04)	‒	‒	‒
Hearing loss degree		0.0241		0.2434
Normal or light	1.04 (1.00, 1.09)	0.0660	1.04 (0.99, 1.09)	0.1008
Moderate to moderately severe	1	‒	1	‒
Severe or complete	1.06 (1.02, 1.11)	0.0064	1.02 (0.96, 1.09)	0.4681
Laterality		<0.0001		0.0024^a^
Unilateral	1.07 (1.04, 1.10)	<0.0001	1.07 (1.02, 1.12)	0.0024^a^
Bilateral	1	‒	1	‒
Hypoacusis time (years)	1.00 (1.00, 1.00)	0.6232	1.00 (1.00, 1.00)	0.6461
Hearing loss onset		0.0324		0.4843
Progressive	1	‒	1	‒
Sudden	1.05 (1.00, 1.09)	0.0324	0.98 (0.93, 1.04)	0.4843
Hearing loss type		0.8012		0.7382
Mixed	1.01 (0.95, 1.06)	0.8012	0.99 (0.94, 1.04)	0.7382
Sensorineural	1	‒	1	‒

a *p* < 0.05.

### Spatial orientation

Hearing loss degree, laterality, presence of comorbidities and tinnitus were significant. Unilateral hearing loss participants also outperformed those with bilateral loss (Table 4).

**Table 4:** Distribution of study variables according to the crude and adjusted average rate as per the Poisson regression model with robust variance and their respective 95% confidence intervals, for the number of correct responses in spatial orientation (n = 134).

Average rate ‒ spatial orientation				
Variable	Non-adjusted (95% CI)	*p*-value	Adjusted (95% CI)	*p*-value
Age	0.99 (0.9, 1.00)	0.0119	‒	‒
At least one comorbidity (SAH, DM or Dyslipidemia)		0.0021		0.0016^a^
No	1	0.0021	1	0.0016^a^
Yes	0.93 (0.88, 0.97)	‒	0.93 (0.88, 0.97)	‒
Smoker		0.7694	‒	‒
No	1	‒	‒	‒
Yes	0.99 (0.92, 1.07)	0.7694	‒	‒
Use of at least one medication (Benzodiazepine, Antidepressants)		0.5011	‒	‒
No	1	‒	‒	‒
Yes	1.02 (0.96, 1.10)	0.5011	‒	‒
Lack of balance		0.1244	‒	‒
No	1	0.1244	‒	‒
Yes	0.94 (0.88, 1.02)	‒	‒	‒
Vertigo		0.0586	‒	‒
No	1	0.0586	‒	‒
Yes	0.94 (0.88, 1.00)	‒	‒	‒
Tinnitus		0.0029		0.0022^a^
No	1	0.0029	1	0.0022^a^
Yes	0.93 (0.88, 0.98)	‒	0.93 (0.88, 0.97)	‒
Hearing loss degree		0.0225		0.0448^a^
Normal or mild	1.07 (1.01, 1.13)	0.0188	1.07 (1.00, 1.12)	0.0353^a^
Moderate or moderately severe	1	‒	1	‒
Severe or complete	1.07 (1.02, 1.13)	0.0116	1.02 (0.95, 1.09)	0.5983
Laterality		0.0109		0.0493^a^
Unilateral	1.06 (1.01, 1.11)	0.0109	1.06 (1.00, 1.12)	0.0493^a^
Bilateral	1	‒	1	‒
Hypoacusis time (years)	1.00 (1.00, 1.00)	0.2827	1.00 (1.00, 1.00)	0.7209
Hearing loss onset		0.0953		0.6815
Progressive	1	‒	1	‒
Sudden	1.05 (0.99, 1.11)	0.0324	0.98 (0.92, 1.06)	0.6815
Hearing loss type		0.0657		0.1397
Mixed	1.05 (1.00, 1.10)	0.0657	1.04 (0.99, 1.10)	0.1397
Sensorineural	1	‒	1	‒

a *p* < 0.05.

### Immediate memory

Hearing loss degree and type impacted immediate memory scores. Normal or mild hearing loss participants had a 7% higher score than those with moderate to moderately severe loss. Those with mixed hearing loss outperformed sensorineural loss individuals (Table 5).

**Table 5:** Distribution of study variables according to the crude and adjusted average rate as per the Poisson regression model with robust variance and their respective 95% Confidence Intervals, for the number of correct responses in immediate memory (n = 134).

Average rate ‒ Immediate Memory				
Variable	Non- adjusted (95% CI)	*p-*value	Adjusted (95% CI)	*p*-value
Age	0.99 (0.9, 1.00)	0.0275	‒	‒
At least one comorbidity (SAH, DM or Dyslipidemia)		0.1569	‒	‒
No	1	0.1569	‒	‒
Yes	0.96 (0.91, 1.02)	‒	‒	‒
Smoker		0.8698	‒	‒
No	1	‒	‒	‒
Yes	1.01 (0.92, 1.10)	0.8698	‒	‒
Use of at least one medication (Benzodiazepine, Antidepressants)		0.2698	‒	‒
No	1	‒	‒	‒
Yes	1.03 (0.98, 1.09)	0.2698	‒	‒
Lack of balance		0.5399	‒	‒
No	1	0.5399	‒	‒
Yes	0.98 (0.93, 1.04)	‒	‒	‒
Vertigo		0.5793	‒	‒
No	1	0.5793	‒	‒
Yes	0.98 (0.93, 1.04)	‒	‒	‒
Tinnitus		0.1513	‒	‒
No	1	0.1513	‒	‒
Yes	0.96 (0.92, 1.01)	‒	‒	‒
Hearing loss degree		0.0400		0.0482^a^
Normal or mild	1.06 (1.01, 1.11)	0.0200	1.07 (1.01, 1.13)	0.0138^a^
Moderate or slightly severe	1	‒	1	‒
Severe or complete	1.02 (0.95, 1.10)	0.5025	1.03 (0.97, 1.10)	0.3415
Laterality		0.5614		0.7374
Unilateral	1.02 (0.96, 1.08)	0.5614	0.99 (0.95, 1.04)	0.7374
Bilateral	1	‒	1	‒
Hypoacusis time (years)	1.00 (1.00, 1.00)	0.3075	1.00 (1.00, 1.00)	0.5938
Hearing loss onset		0.9814		0.9132
Progressive	1	‒	1	‒
Sudden	1.00 (0.92, 1.08)	0.9814	1.00 (0.93, 1.07)	0.9132
Hearing loss type		0.0002		0.0007
Mixed	1.07 (1.03, 1.12)	0.0002	1.08 (1.03, 1.13)	0.0007
Sensorineural	1	‒	1	‒

a *p* < 0.05.

### Attention and calculation

Unilateral hearing loss, absence of vertigo and absence of tinnitus were associated with better performance. Participants with unilateral hearing loss had a 77% higher score than those with bilateral loss. Additionally, the average number of correct responses decreases by 1% for each year of age (*p* = 0.0411) (Table 6).

**Table 6:** Distribution of study variables according to the crude and adjusted average rate as per the Poisson regression model with robust variance and their respective 95% Confidence Intervals, for the number of correct responses for attention and calculation (n = 134).

Average rate ‒ Attention and Calculation				
Variable	Non-adjusted (95% CI)	*p*-value	Adjusted (95% CI)	*p*-value
Age	0.99 (0.98, 1.00)	0.1125	0.99 (0.98, 1.00)	0.0411^a^
At least one comorbidity (SAH, DM or Dyslipidemia)		0.3928		‒
No	1	‒	‒	‒
Yes	0.88 (0.67, 1.18)	0.3928	‒	‒
Smoker		0.0806	‒	‒
No	1	‒	‒	‒
Yes	0.69 (0.45, 1.05)	0.0806	‒	‒
Use of at least one medication (benzodiazepines or antidepressants)		0.2015	‒	‒
No	1	0.2015	‒	‒
Yes	0.73 (0.45, 1.19)	‒	‒	‒
Lack of balance		0.3155	‒	‒
No	1	0.3155	‒	‒
Yes	0.85 (0.63, 1.16)	‒	‒	‒
Vertigo		0.0221		0.0080^a^
No	1	0.0221	1	0.0080^a^
Yes	0.70 (0.51, 0.95)	‒	0.68 (0.51, 0.90)	‒
Tinnitus		0.0215^a^	‒	‒
No	1	0.0215^a^	‒	‒
Yes	0.73 (0.56, 0.95)	‒	‒	‒
Hearing loss degree		0.0082		0.7538
Normal or mild	1.26 (0.91, 1.76)	0.1635	1.13 (0.82, 1.57)	0.4522
Moderate or slightly severe	1	‒	1	‒
Severe or complete	1.62 (1.19, 2.20)	0.0021	1.15 (0.74, 1.81)	0.8589
Laterality		0.0119		0.0396^a^
Unilateral	1.47 (1.09, 1.99)	0.0119	1.77 (1.03, 3.04)	0.0396^a^
Bilateral	1	‒	1	‒
Hypoacusis time (years)	1.00 (0.99, 1.01)	0.9205	1.00 (0.99, 1.01)	0.9372
Hearing loss onset		0.3528		0.3126
Progressive	1	‒	1	‒
Sudden	1.23 (0.79, 1.90)	0.3528	0.79 (0.45, 1.37)	0.3126

a *p* < 0.05.

### Evocation memory

Hearing loss onset, the absence of comorbidities and the absence of tinnitus were significant. Progressive hearing loss participants performed better than those with sudden onset. Absence of comorbidities and tinnitus also led to better scores (Table 7).

**Table 7:** Distribution of study variables according to the crude and adjusted average rate as per the Poisson regression model with robust variance and their respective 95% Confidence Intervals, for the number of correct responses in evocation memory (n = 134).

Average rate ‒ Evocation Memory				
Variable	Non-adjusted (95% CI)	*p-*value	Adjusted (95% CI)	*p-*value
Age	0.99 (0.98, 1.00)	0.0424	‒	‒
At least one comorbidity (SAH, DM or Dyslipidemia)		0.0670		0.0298^a^
No	1	‒	1	‒
Yes	0.83 (0.67, 1.01)	0.0670	1.26 (1.02, 1.54)	0.0298^a^
Smoker		0.8368	‒	‒
No	1	‒	‒	‒
Yes	0.97 (0.73. 1.29)	0.8368	‒	‒
Use of at least one medication (benzodiazepines or antidepressants)		0.5771	‒	‒
No	1	0.5771	‒	‒
Yes	0.91 (0.65, 1.27)	‒	‒	‒
Lack of balance		0.1125	‒	‒
No	1	0.1125	‒	‒
Yes	0.83 (0.66, 1.04)	‒	‒	‒
Vertigo		0.1379	‒	‒
No	1	0.1379	‒	‒
Yes	0.85 (0.68, 1.05)	‒	‒	‒
Tinnitus		0.0205		0.0170^a^
No	1	0.0205	1	0.0170^a^
Yes	0.78 (0.63, 0.96)	‒	0.78 (0.64, 0.96)	‒
Hearing loss degree		0.3887		0.4033
Normal or Mild	1.15 (0.89, 1.48)	0.2873	1.18 (0.92, 1.52)	0.1853
Moderate or slightly severe	1	‒	1	‒
Severe or complete	1.15 (0.91, 1.45)	0.2415	1.01 (0.73, 1.40)	0.9593
Laterality		0.2338		0.0883
Unilateral	1.15 (0.91, 1.46)	0.2338	1.38 (0.95, 1.99)	0.0883
Bilateral	1	‒	1	‒
Hearing loss onset		0.2021		0.0101^a^
Progressive	1.16 (0.89, 1.56)	0.3085	1.54 (1.11, 2.15)	0.0101^a^
Sudden	1	‒	1	‒
Hearing loss type		0.2021		0.6852
Mixed	1.16 (0.92, 1.45)	0.2021	1.05 (0.83, 1.32)	0.6852
Sensorineural	1	‒	1	‒

a *p* < 0.05.

### Language

Participants with unilateral loss scored 14% higher than those with bilateral hearing loss. Patients with severe or complete hearing loss scored 8% lower than those with moderate or moderately severe hearing loss. Other significant factors included absence of comorbidities, using of medication and absence of vertigo. Additionally, the average number of correct responses in language decreases by 1% for each one-year increase in age (Table 8).

**Table 8:** Distribution of study variables according to the crude and adjusted average rate as per the Poisson regression model with robust variance and their respective 95% Confidence Intervals, for the number of correct responses in language (n = 134).

Average rate ‒ Language				
Variable	Non-adjusted (95% CI)	*p*-value	Adjusted (95% CI)	*p*-value
Age	0.99 (0.98, 1.00)	<0.0001	0.99 (0.98, 1.00)	<0.0001^a^
At least one comorbidity (SAH, DM or Dyslipidemia)		0.0005^a^		‒
No	1	‒	‒	‒
Yes	0.90 (0.85, 0.95)	0.0005^a^	‒	‒
Smoker		0.0418^a^	‒	‒
No	1	0.0418^a^	‒	‒
Yes	0.88 (0.78, 1.00)	‒	‒	‒
Use of at least one medication (Benzodiazepine, Antidepressants)		0.0154		0.0079^a^
No	1	‒	1	‒
Yes	1.08 (1.01, 1.14)	0.0154	1.10 (1.02, 1.18)	0.0079^a^
Lack of balance		0.9018	‒	‒
No	1	0.9018	‒	‒
Yes	1.00 (0.93, 1.06)	‒	‒	‒
Vertigo		0.0432^a^	‒	0.0189^a^
No	1	0.0432^a^	1	0.0189^a^
Yes	0.93 (0.86, 1.00)	‒	0.92 (0.85, 0.99)	‒
Tinnitus		0.7431	‒	‒
No	1	0.7431	‒	‒
Yes	0.99 (0.91, 1.06)	‒	‒	‒
Hearing loss degree		0.0047		0.3002
Normal or mild	1.04 (0.94, 1.15)	0.3952	1.01 (0.92, 1.10)	0.8617
Moderate or moderately severe	1	‒	1	‒
Severe or complete	1.12 (1.04, 1.19)	0.0011	0.92 (0.86, 0.99)	0.0223^a^
Laterality		<0.0001		<0.0001^a^
Unilateral	1.15 (1.10, 1.21)	<0.0001	1.14 (1.10, 1.21)	<0.0001^a^
Bilateral	1	‒	1	‒
Hearing loss onset		<0.0001		0.8641
Progressive	1	‒	1	‒
Sudden	1.12 (1.07, 1.19)	<0.0001	1.01 (0.93, 1.09)	0.8641
Hearing loss type		0.2807		0.7548
Mixed	1.04 (0.97, 1.11)	0.2807	0.99 (0.93, 1.05)	0.7548
Sensorineural	1	‒	1	‒

a *p* < 0.05.

## Discussion

In this study, we evaluated general performance on the MMSE. We also analyzed an aspect that has not yet been explored in the literature: performance in each of the six cognitive subdomains that make up the MMSE, separately.

Several studies in the last few years have been clearly addressing the hearing loss/cognitive impairment association. However, the pathophysiological mechanisms involved in this association remain poorly explored. Cognitive domains affected by hearing loss, at higher or lower degree, are not yet established. Gates (1996) [15] and Räihä (2001) [25] showed association between hearing loss and worse MMSE performance. However, these studies did not detail which characteristics inherent to hearing loss would be associated with a greater impact on the MMSE score.

MMSE was chosen for the cognitive assessment because it does not demand minimum schooling from tested individuals, and it has a validated version for Brazilian Portuguese. Only 14.18% of participants (Table 1) declared to have their hearing limitation impairing their ability to answer to MMSE. Powell (2022) [12], Nichols et al. (2022) [16] and Bott (2019) [26] also showed such small impact on sensory impairment in the cognitive assessment.

In this study, individuals over 18 years old were assessed. There was vast majority of older adults (61% of participants were 60 years old or older), but the sample was not limited to this population, as most studies available in the literature. We found that age had no influence on general MMSE score, but it was related to lower averages of correct responses in two cognitive domains: Attention/Calculation and Language.

### Overall MMSE performance

Among individuals complaining of imbalance, there was 43% higher changes of having general MMSE performance below normal based on schooling in comparison to those without it (PR 1.43 [1.07–1.92] 95% CI, *p* = 0.0167 – Table 2). This finding meets the association that has also been pointed out in the literature in the last few years between cognitive performance and balance disorders.

We also observed that chances of having overall MMSE performance below normal based on schooling was 53% higher in individuals who had sudden hearing loss onset than in those who had progressive onset of it (PR 1.53 [1.00–2.36] 95% CI, *p* = 0.0468 – Table 2).

There is little literature evaluating associations between sudden hearing loss and cognitive impairment. Tai (2021) assessed a retrospective cohort and found close association between sudden hearing loss and increased risk of developing dementia [27]. It was not possible finding studies that had analyzed the association between sudden hearing loss and other forms of cognitive impairment, as well as studies that have specifically assessed the performance of sudden deafness carriers in the MMSE. It is important highlighting that datum about sudden deafness may have been influenced by its collection method: hearing loss onset type was classified based on self-reported information.

There was no significant association with any other variable linked to general MMSE performance below normal based on schooling. However, the literature points out the influence of other variables related to hearing loss. This influence was found in the present study just when each cognitive domain was analyzed in separate.

### Cognitive domains

In the current study, unilateral hearing loss accounted for higher rates of correct answers than bilateral loss in four different cognitive domains: spatial orientation, time orientation, attention and calculation, and language. The population-based Korean study published by Lee (2021) showed significant increase in hearing loss effect on the Korean population’s cognitive functions. Individuals with bilateral hearing loss had worse performance than those with unilateral loss [27]. Fritze (2016) carried out a prospective study and found that bilateral hearing loss was related to increase by 43% in the risk of developing dementia and that unilateral hearing loss was associated with 20% increase in this same risk [28]. Results in the current study did not evidence differences between unilateral and bilateral hearing loss carriers when it comes to higher risk of general MMSE performance below normal based on schooling. However, there were significant differences in the number of correct answers recorded for four of the six MMSE domains.

Mild hearing loss carriers accounted for higher rates of correct answers in comparison to moderate to moderately severe hearing loss carriers in two domains: spatial orientation and immediate memory. Regarding hearing loss degree, Tay (2006) pointed out that adult individuals suffering with moderate to worst-degree hearing loss (tetratonal average in the best ear > 40 dB) presented mean general MMSE scoring slightly lower than individuals without it (28.1 × 28.7, *p* < 0.001) [29]. Therefore, this study did not show differences among moderate, severe, profound or complete hearing loss. Kopper (2009) identified that older adults with normal hearing or with mild hearing loss in the best ear had better MMSE performance than those with moderate or severe loss [30]. There was no record of profound or complete hearing loss. Lin (2011) has shown that relative risk (95% CI) for dementia incidence in comparison to normal hearing was 1.89 (1.00–3.58) for mild degree hearing loss, 3.00 (1.43–6.30) for moderate loss and 4.94 (1.09–22.40) for severe hearing loss [31]. Jupiter (2012) assessed the MMSE performance of institutionalized older adults and found better performance among mild hearing loss carriers in comparison to those carrying moderate or severe loss [32]. However, the most severe hearing losses were pooled into a single group.

The present results have shown the best performance recorded for the group of participants suffering with mild hearing loss in the best ear in comparison to the group with moderate/moderately severe loss only in domains ‘spatial orientation’ (7% higher, *p* = 0.0353) and ‘immediate memory’ (7% higher, *p* = 0.0138). There was no significant difference between these groups in the other cognitive domains and in general MMSE scoring. Yet, there was no significant difference in comparison to the group with severe/profound/complete hearing loss. Assumingly, such a finding is related to the small number of individuals suffering with severe/profound/complete hearing loss in the present sample. These outcomes are similar to those published by Mattiazzi (2016), who also did not find significant differences in general MMSE scoring between groups with different hearing loss degrees, although there was significant difference in this variable in cognitive domain ‘language’ [33].

Those carrying mixed hearing loss recorded higher rates of correct answers in comparison to the ones with sensorineural loss in the immediate memory cognitive domain. Assumingly, this finding can be associated with the auditory nature of this test: individuals have to hear three words in sequence, which are said by the appraiser, and they have to repeat these words right away. There was no significant difference in the other domains.

Individuals with progressive hearing loss onset recorded the highest rates of correct answers in the evocation memory domain in comparison to those who recorded sudden loss onset. Therefore, history of sudden hearing loss onset was associated with bigger changes of recording general MMSE performance below normal based on schooling and of lower scoring in this specific cognitive domain. As previously highlighted, the literature lacks information about the sudden deafness/cognitive impairment association.

Individuals without comorbidities recorded the highest rates of correct answers in comparison to those with at least one comorbidity in four cognitive domains: time orientation, spatial orientation, evocation memory and language. These findings meet the literature, because the assessed comorbidities (high blood pressure, diabetes mellitus or dyslipidemia) are independent risk factors for cognitive impairment [2].

Regarding hearing loss associated symptoms, individuals without tinnitus complaint recorded higher rates of correct answers in comparison to those without it in three domains: spatial orientation, attention and calculation, and evocation memory. This outcome is relevant and seems to follow the literature on the likely interference of tinnitus with cognitive functions, such as attention and memory [34]. Individuals denying vertigo complaint recorded the highest rate of correct answers in cognitive domains “language” and “attention and calculation” in comparison to those complaining of it. This finding also seems to meet the association set in the literature between cognitive functions and balance disorders [35].

The present study has some methodological limitations because it is a cross-sectional observation study. There are also limitations regarding the collection of some data gathered from information in medical reports or from participants’ self-referenced information. Yet, it is important mentioning the small number of individuals with severe, profound or complete hearing loss in the sample, although this finding was expected, because the herein assessed sample comprised a population recommended for hearing-aid use ‒ therefore, moderate losses are more frequent in this group.

Despite the limitations, the present survey provided new and relevant data. Most studies available in the literature have used MMSE to assess the cognition of hearing loss carriers and only analyzed general scoring in the questionnaire. However, the current survey allowed assessing the impact of each cognitive domain, in separate. Besides, the herein adopted criteria to diagnose and classify hearing losses were objective, all patients were subjected to pure tone audiometry.

Besides audiometric data, it was possible assessing clinical data, such as comorbidities, tinnitus complaint and body balance changes, which are related to hearing loss and to its impact on cognition.

In-depth studies comprising a larger number of hearing loss carriers at different types and degrees of it must be carried out in order to find the hearing impairment features related to higher risk of developing cognitive impairment. This information will help better understanding the pathophysiology of the hearing loss/cognitive impairment association. It will also help establishing better prevention and treatment strategies and policies either for hearing loss or for cognitive impairment and dementia.

## Conclusion

The present results have confirmed that differences in features inherent to hearing loss are associated with the negative impact of cognitive performance.

Complaints of lack of balance and sudden hearing loss onset appeared to be associated with higher risk of performance in the mini mental state exam below normal, based on individuals’ schooling.

Although bilateral sensorineural hearing loss of moderate to moderately severe degree, presence of tinnitus, vertigo and comorbidities were factors without significant impact on general MMSE performance, they were associated with worse scores in specific cognitive domains in the questionnaire. Bilateral hearing loss, tinnitus and vertigo complaints had relevant negative impact on the attention and calculation domain, whereas sudden loss had quite relevant impact on the evocation memory domain in comparison to progressive hearing loss.

## Funding

We hereby declare that the research project titled “IMPACT OF HEARING IMPAIRMENT ON COGNITIVE PERFORMANCE” was conducted without any external financial support or sponsorship. We confirm that all expenses associated with the execution of this research project were personally covered, including but not limited to, research materials, equipment, travel expenses, and any other costs related to the project.

This declaration serves to affirm that no funding, grants, or sponsorship were received from any public, private, or non-profit organizations, entities, or individuals. The independence of the research has been maintained throughout the project, ensuring that the findings and conclusions drawn are free from any external influence or bias.

## Declaration of competing interest

We hereby declare that there are no conflicts of interest, financial or otherwise, that could have influenced the work presented in this manuscript titled “IMPACT OF HEARING IMPAIRMENT ON COGNITIVE PERFORMANCE”. This encompasses, but is not limited to, employment, consultancies, stock ownership, honoraria, expert testimony, patents or patent applications, and grants or other funding.

## References

[1] Chadha S., Kamenov K., Cieza A. (2021). The world report on hearing, 2021. Bull World Health Organ..

[2] Livingston G., Sommerlad A., Orgeta V., Costafreda S.G., Huntley J., Ames D. (2017). Dementia prevention, intervention, and care. Lancet..

[3] Prince M., Wimo A., Guerchet M., Gemma-Claire M., Wu Y.-T., Prina M. World Alzheimer Report 2015 The Global Impact of Dementia an AnAlysIs of PrevAlence, IncIDence, CosT AnD TrenDs. www.alz.co.uk/worldreport2015corrections.

[4] Harada C.N., Natelson Love M.C., Triebel K.L. (2013). Normal cognitive aging. Clin Geriatr Med..

[5] Powell D.S., Oh E.S., Lin F.R., Deal J.A. (2021). Hearing impairment and cognition in an aging world. J Assoc Res Otolaryngol..

[6] Zheng Y., Fan S., Liao W., Fang W., Xiao S., Liu J. (2017). Hearing impairment and risk of Alzheimer’s disease: a meta-analysis of prospective cohort studies. Neurol Sci..

[7] Liang Z., Li A., Xu Y., Qian X., Gao X. (2021). Hearing loss and dementia: a meta-analysis of prospective cohort studies. Front Aging Neurosci..

[8] Gurgel R.K., Ward P.D., Schwartz S., Norton M.C., Foster N.L., Tschanz J.T. (2014). Relationship of hearing loss and dementia: a prospective, population-based study. Otol Neurotol..

[9] Lee H.J., Joo Y.H., Do Han K., Park K.H. (2021). Association between hearing loss and cognitive disorder: A nationwide population-based study. Yonsei Med J..

[10] Loughrey D.G., Kelly M.E., Kelley G.A., Brennan S., Lawlor B.A. (2018). Association of age-related hearing loss with cognitive function, cognitive impairment, and dementia a systematic review and meta-analysis. JAMA Otolaryngol Head Neck Surg..

[11] Wayne R.V., Johnsrude I.S. (2015). A review of causal mechanisms underlying the link between age-related hearing loss and cognitive decline. Ageing Res Rev..

[12] Powell D.S., Oh E.S., Reed N.S., Lin F.R., Deal J.A. (2022). Hearing loss and cognition: what we know and where we need to go. Front Aging Neurosci.

[13] (2021). https://youtu.be/EmXwAnP9puQ.

[14] Tong J., Zhang J., Xu L., Liu M., Min J., Yao M. (2022). Effect of hearing loss on cognitive function in patients with mild cognitive impairment: A prospective, randomized, and controlled study. Front Aging Neurosci..

[15] Gates G.A., Cogg J.L., Linn R.T., Rees T., Wolf P.A., D’Agostino R.B. (1996). Central auditory dysfunction, cognitive dysfunction, and dementia in older people. Arch Otolaryngol Head Neck Surg..

[16] Nichols E., Deal J.A., Swenor B.K., Abraham A.G., Armstrong N.M., Carlson M.C. (2022). Assessing Bias in Cognitive Testing for Older Adults with Sensory Impairment: An Analysis of Differential Item Functioning in the Baltimore Longitudinal Study on Aging (BLSA) and the Atherosclerosis Risk in Communities Neurocognitive Study (ARIC-NCS). J Int Neuropsychol Soc..

[17] Rutherford B.R., Brewster K., Golub J.S., Kim A.H., Roose S.P. (2018). Sensation and psychiatry: linking age-related hearing loss to late-life depression and cognitive decline. Am J Psychiatry.

[18] Simonsick E.M., Kasper J.D., Phillips C.L. (1998). Physical disability and social interaction: factors associated with low social contact and home confinement in disabled older women (The Women’s Health and Aging Study). J Gerontol B Psychol Sci Soc Sci..

[19] Füllgrabe C. (2020). On the possible overestimation of cognitive decline: the impact of age-related hearing loss on cognitive-test performance. Front Neurosci..

[20] Guerreiro M.J.S., Van Gerven P.W.M. (2017). Disregarding hearing loss leads to overestimation of age-related cognitive decline. Neurobiol Aging..

[21] Armstrong N.M., An Y., Doshi J., Erus G., Ferrucci L., Davatzikos C. (2019). Association of Midlife hearing impairment with late-life temporal lobe volume loss. JAMA Otolaryngol Head Neck Surg..

[22] Peelle J.E. (2018). Listening effort: how the cognitive consequences of acoustic challenge are reflected in brain and behavior. Ear Hear..

[23] World Health Organization (2020). https://www.who.int/publications/i/item/9789240001480.

[24] Brucki S.M.D., Nitrini R., Caramelli P., Bertolucci P.H.F., Okamoto I.H. (2003). Sugestões para o uso do mini-exame do estado mental no Brasil. Arq Neuropsiquiatr..

[25] Räihä I., Isoaho R., Ojanlatva A., Viramo P., Sulkava R., Kivelä S.L. (2001). Poor performance in the Mini-Mental State Examination due to causes other than dementia. Scand J Prim Health Care..

[26] Bott A., Meyer C., Hickson L., Pachana N.A. (2019). Can adults living with dementia complete pure-tone audiometry? A systematic review. Int J Audiol..

[27] Tai S.Y., Shen C.T., Wang L.F., Chien C.Y. (2021). Association of sudden sensorineural hearing loss with dementia: a nationwide cohort study. BMC Neurol..

[28] Fritze T., Teipel S., Óvári A., Kilimann I., Witt G., Doblhammer G. (2016). Hearing impairment affects dementia incidence. An analysis based on longitudinal health claims data in Germany. PLoS One..

[29] Tay T., Jie J.W., Kifley A., Lindley R., Newall P., Mitchell P. (2006). Sensory and cognitive association in older persons: findings from an older Australian population. Gerontology..

[30] Original A., Kopper H., Ribeiro Teixeira A., Dorneles S. (2009). Cognitive performance of a group of elders: influence of hearing, age, sex, and education. Arq Int Otorrinolaringol / Intl Arch Otorhinolaryngol..

[31] Lin F.R., Metter E.J., O’Brien R.J., Resnick S.M., Zonderman A.B., Ferrucci L. (2011). Hearing loss and incident dementia. Arch Neurol..

[32] Jupiter T. (2012). Cognition and screening for hearing loss in nursing home residents. J Am Med Dir Assoc..

[33] Mattiazzi A., Gresele A., Henning T., Costa M. (2016). Resultados do miniexame do estado. Estudos Interdisciplinares Sobre o Envelhecimento..

[34] Tavanai E., Mohammadkhani G. (2018). A different view on the link between tinnitus and cognition; is there a reciprocal link?. Int J Neurosci..

[35] Xie D., Welgampola M.S., Miller L.A., Young A.S., D’Souza M., Breen N. (2022). Subjective cognitive dysfunction in patients with dizziness and vertigo. Audiol Neurotol..

